# Association among PlA1/A2 gene polymorphism, laboratory aspirin resistance and clinical outcomes in patients with coronary artery disease: An updated meta-analysis

**DOI:** 10.1038/s41598-019-49123-y

**Published:** 2019-09-11

**Authors:** Jing Wang, Jie Liu, Yaqing Zhou, Fei Wang, Ke Xu, Deyu Kong, Jianling Bai, Jun Chen, Xiaoxuan Gong, Haoyu Meng, Chunjian Li

**Affiliations:** 1 Departments of Cardiology, First Affiliated Hospital of Nanjing Medical University, Nanjing, Jiangsu China; 2 Departments of Cardiology, The Affiliated Huaian No.1 People’s Hospital of Nanjing Medical University, Huaian, Jiangsu China; 3 Departments of Cardiology, Changzhou No. 2 People’s Hospital Affiliated with Nanjing Medical University, Changzhou, Jiangsu China; 4 Department of Cardiology, Xuzhou Children’s Hospital, Xuzhou Medical University, Xuzhou, Jiangsu China; 5 Department of Cardiology, Jining First People’s Hospital, Jining, Shandong China; 6 Department of Epidemiology and Biostatistics, School of Public Health, Nanjing Medical University, Nanjing, Jiangsu China; 7 Departments of Cardiology, Maanshan People’s Hospital, Maanshan, Anhui China

**Keywords:** Cardiovascular genetics, Platelets

## Abstract

The aim of this study was to investigate the association among the PlA1/A2 gene polymorphism, laboratory aspirin resistance and adverse clinical outcomes in coronary artery disease (CAD) patients who were on aspirin maintainance therapy. A comprehensive literature search was performed and 35 eligible clinical trials including 19025 CAD patients were recruited. Adverse clinical outcomes involving all-cause death, non-fatal myocardial infarction (MI), ischemic stroke and target vessel revascularization (TVR) were analyzed. The definition of aspirin resistance in each study was accepted. Meta-analysis was performed using the Review Manager 5.3.5 System. In CAD patients, the PlA2 gene carriers had similar incidence of laboratory aspirin resistance compared to those with PlA1/A1 genotype [29.7% vs 28.3%, OR = 0.94 (95% CI 0.63 to 1.40, P = 0.74)], and there were no significant differences in the adverse clinical outcomes between the PlA2 carriers and the PlA1/A1 genotype patients. However, the laboratory aspirin non-responders had higher risks of death [7.9% vs. 2.5%, OR = 2.42 (95% CI 1.86 to 3.15, P < 0.00001)] and TVR [4.5% vs. 1.7%, OR = 2.20 (95% CI 1.19 to 4.08, P = 0.01)] compared to the responders. In aspirin-treated CAD patients, the laboratory aspirin resistance predicts all-cause death and TVR. However, the PlA1/A2 gene polymorphism predicts neither the laboratory aspirin response nor the clinical outcomes.

## Introduction

Aspirin (acetylsalicylic acid) is a well-known baseline anti-platelet agent for the treatment and prevention of coronary artery disease (CAD). It irreversibly acetylates a serine residue at position 529 in platelet prostaglandin synthase, and inhibits cyclooxygenase (COX) channel associated with platelet aggregation. However, up to 24% patients were reported to be resistant to aspirin^1^. This mechanism of resistance and its clinical impact are under investigation.

PlA1/A2 polymorphism, a single nucleotide substitution (T → C) at position 1565 in exon 2 of the GP IIIa (a component of the final platelet aggregation pathway GPIIb/IIIa) gene has been reported to be associated with the laboratory detected aspirin resistance^2–5^ and adverse clinical outcomes^6–9^. However, the results are inconsistent among different studies^10–12^. In addition, study results regarding whether aspirin resistance is associated with adverse cardiovascular events are also inconsistent^13–17^.

This meta-analysis aimed to include the latest studies and update the concept regarding whether the PlA1/A2 gene polymorphism predicts laboratory aspirin resistance and/or cardiovascular outcomes, and whether laboratory detected aspirin resistance predicts cardiovascular outcomes in CAD patients who are on aspirin treatment. The results of this study will provide evidence for the individualized anti-platelet treatment.

## Materials and Methods

### Eligibility and search strategy

We performed a comprehensive literature search to identify all the studies investigating the association among PlA1/A2 gene polymorphism, aspirin resistance and cardiovascular outcomes in patients with coronary artery disease treated with aspirin. The literature was scanned by computerized searches of Pubmed, Embase, Cochrane library and Chinese Medical Journal Network databases from establishment to September 2018. The search strategy included a combination of medical subject headings and text words as follows: *A* = *(aspirin OR acetylsalicylic acid), B* = *(platelet aggregation OR platelet activity OR aspirin resistance OR aspirin non-responder OR aspirin low response), C* = *(gene OR polymorphism OR mutation OR genotype OR allele OR genetic), D* = *(death OR stroke OR myocardial infarction OR revascularization)*. The Medical Subject Headings terms and text words A, B, C were used for the search to investigate the association between PlA1/A2 gene polymorphism and aspirin resistance; The Medical Subject Headings terms and text words A, C, D were used for the search to assess the association between PlA1/A2 gene polymorphism and cardiovascular outcomes; The Medical Subject Headings terms and text words A, B, D were used for the search to investigate the association between aspirin resistance and cardiovascular outcomes. Reference literatures of the appropriate trials were hand searched. The search results were limited to human. No language restriction was enforced.

### Study selection

**Figure 1:**
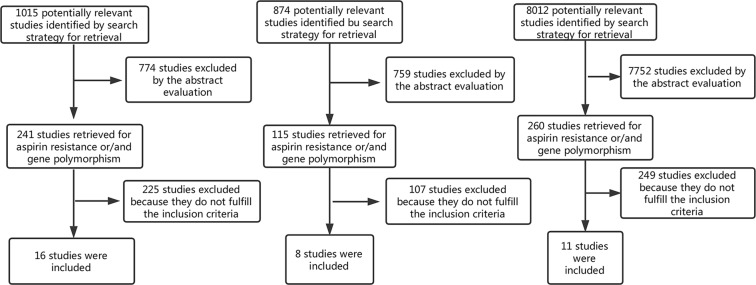
Flowchart of the study selection.

The inclusion criteria of this study include: (1) studies that include patients with confirmed CAD; (2) studies that include patients who were treated with aspirin for secondary prevention of cardiovascular events; (3) studies that contain a clear description of the method used to establish the effects of aspirin on platelet reactivity; (4) studies that contain a clear description of the PlA1/A2 polymorphism; (5) studies that report the incidence of either death, myocardial infarction (MI), ischemic stroke, or target vessel revascularization (TVR). Studies that meet the criteria (1), (2), (3), (4) were adopted to analyze the relationship between PlA1/A2 gene polymorphism and aspirin resistance. Studies that meet criteria (1), (2), (4), (5) were adopted to analyze the relationship between PlA1/A2 gene polymorphism and cardiovascular outcomes. Studies that meet criteria (1), (2), (3), (5) were adopted to analyze the relationship between aspirin resistance and cardiovascular outcomes (Fig. 1). We assessed the included observational studies according to the Newcastle-Ottawa Scale.

### Clinical outcomes

The adverse clinical outcomes involve all-cause death, non-fatal MI, ischemic stroke and TVR. The definition of each event in the original articles was accepted.

### Statistical analysis

Statistical analysis was performed using Review Manager 5.3.5 (The Cochrane Collaboration, Oxford, England). The odds ratio (OR) or relative risk (RR), and 95% confidence interval (CI) for categorical variables were calculated using a fixed-effect model with the Mantel-Haenszel method. The DerSimonian and Laird random effect model was applied to the calculated OR and RR in case of significant heterogeneity across studies. Statistical heterogeneity was evaluated using the Q statistic with P < 0.1. Points were evaluated at the longest follow-up available. Statistical significance was considered as P < 0.05.

## Results

### PlA1/A2 gene polymorphism and aspirin resistance

**Figure 2:**
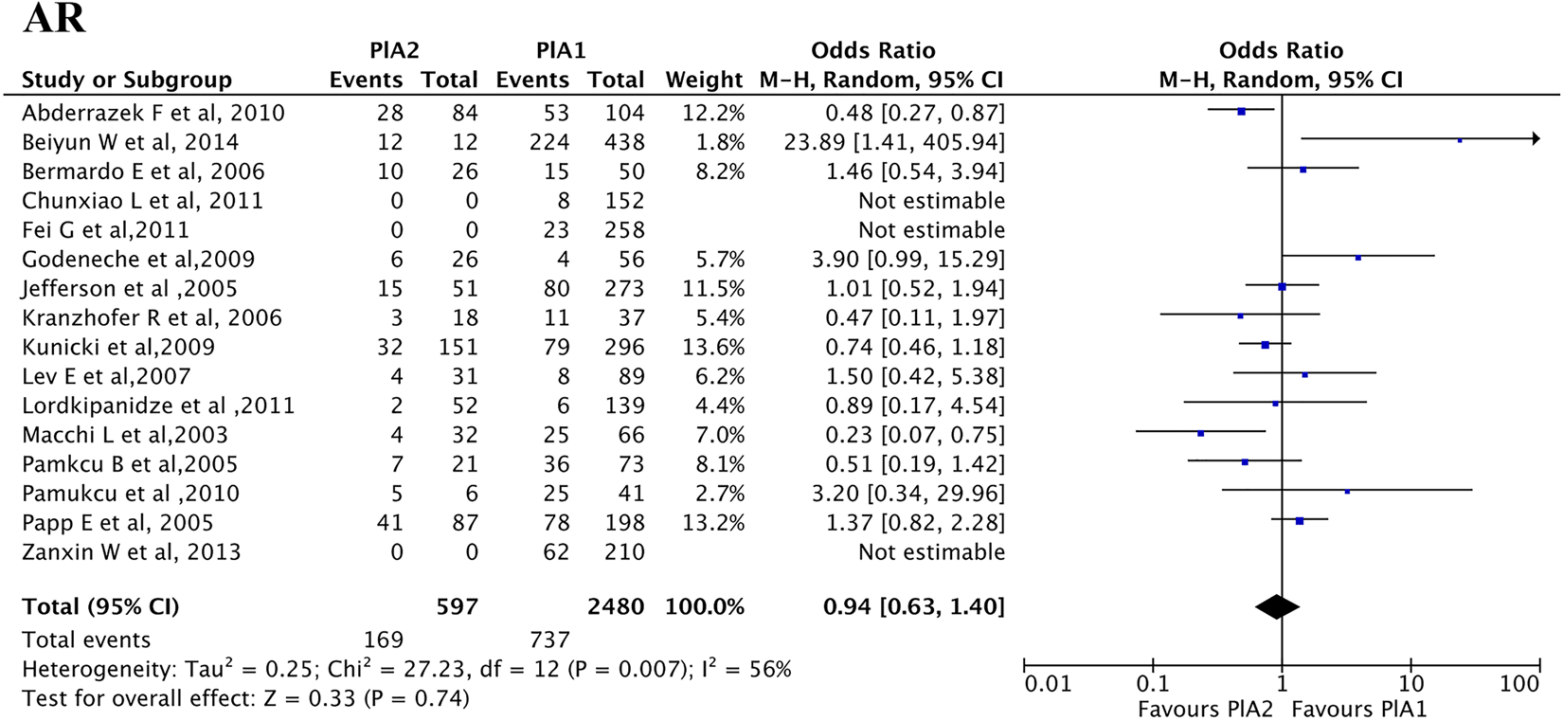
Association between PlA1/A2 polymorphism and laboratory aspirin resistance. The position of the blue squares corresponds to the odds ratio (OR) per study and the horizontal black line to the 95% confidence intervals (CI). The size of the square is proportional to the relative weight of that study w(%) to compute the overall OR (black diamond). The width of the diamond represents the 95% CI of the overall OR. If a 95% CI spans one (indicated by the black vertical solid line), this study has found no significant difference in the incidence of aspirin resistance (AR) between patients carrying the PlA1 and PlA2 alleles. This meta-analysis shows no significant change in the incidence of AR of patients carrying the PlA2 allele over those carrying PlA1 allele. The overall OR is 0.94 (P = 0.74). Note that the p-value mentioned here is the p-value for Z test.

Sixteen studies^18–33^ including 3077 CAD patients were selected to assess the association between PlA1/A2 gene polymorphism and aspirin resistance. Details of included studies are summarized in Table 1. Heterogeneity testing showed considerable bias between different studies (P = 0.004), so a random effect model was adopted. An OR of 0.94 (95% CI 0.63 to 1.40, P = 0.74) was observed for aspirin resistance in patients carrying the PlA2 allele (PlA1/A2 + PlA2/A2) (Fig. 2).

**Table 1:** Characteristics of eligible studies referring to the association between PlA1/A2 gene polymorphism and aspirin resistance.

Source	Number of patients	Study design	Aspirin dosage (mg)	Method of assessment of lab AR	Definition of assessment of lab AR	PlA2 (AR/AS)	PlA1 (AR/AS)
Abderrazek F *et al*., 2010	188	Cohort study	250	PFA-100	CEPI-CT < 160 s	28/56	53/51
Beiyun W *et al*., 2014	450	Cohort study	100	LTA	PL_ADP_ ≥ 70% and PL_AA_ ≥ 20%	12/0	224/214
Bernardo E *et al*., 2006	76	Cohort study	100	PFA-100	CEPI-CT < 193s	10/16	15/35
Chunxiao L, *et al*., 2011	152	Cohort study	100	LTA	PL_ADP_ ≥ 70% and PL_AA_ ≥ 20%	0/0	8/144
Fei G *et al*., 2011	258	Cohort study	100	11-DH-TXB_2_	11-DH-TXB_2_ ≥ 20%	0/0	23/235
Kranzhofer R *et al*., 2006	55	Cohort study	100	LTA	PL_ADP_ ≥ 70% and PL_AA_ ≥ 20%	3/15	11/26
Lev EI *et al*., 2007	120	Cohort study	325	LTA	PL_ADP_ ≥ 70% and PL_AA_ ≥ 20%	4/27	8/81
Macchi L *et al*., 2003	98	Cohort study	160	PFA-100	CEPI-CT < 186 s	4/28	25/41
Pamukcu B *et al*., 2005	94	Cohort study	100–300	PFA-100	CEPI-CT < 186 s	7/14	36/37
Papp E *et al*., 2005	285	Case-control study	100–325	LTA	UK	41/46	78/120
Zanxin W *et al*., 2013	210	Cohort study	100	11-DH-TXB_2_	11-DH-TXB_2_ ≥ 20%	0/0	62/148
Godeneche *et al*., 2009	82	Cohort study	160	PFA-100	CEPI-CT < 187 s	6/26	4/56
Jefferson *et al*., 2005	324	Cohort study	81	LTA	PL_ADP_ ≥ 70% and PL_AA_ ≥ 20%	15/51	80/273
Kunicki *et al*., 2009	447	Cohort study	75–150	PFA-100	CEPI-CT < 164 s/192 s	32/151	79/296
Lordkipanidze *et al*., 2011	191	Cohort study	85–325	LTA	PL_ADP_ ≥ 70% and PL_AA_ ≥ 20%	2/52	6/139
Pamukucu *et al*., 2010	47	Cohort study	193/207	PFA-100	CEPI-CT < 186 s	5/6	25/41

AR, aspirin resistance; AS, aspirin sensitive; PFA-100, platelet function analyzer-100; ADP, adenosine diphosphate; AA, arachidonic acid; PL_ADP_, ADP-induced platelet aggregation; PL_AA_, AA-induced platelet aggregation; UK, unknown;11-DH-TXB_2_, 11-dehydro-thromboxane B_2_; LTA, light transmittance aggregometry; CEPI-CT, collagen epinephrine-close time.

A subgroup analysis was conducted considering that the methodology used to assess aspirin resistance might influence the association. The data available allowed the comparison of two methods: light transmission aggregometry (LTA) and point-of-care assay PFA-100 (Supplemental Figs 1 and 2).

The LTA subgroup analysis showed significant homogeneity between studies (P = 0.22), but did not reveal a significant association between carriage of the PLA2 allele and aspirin resistance (OR 1.35, 95% CI 0.96, 1.91; P = 0.09). The PFA-100 subgroup analysis revealed a significant association between carriage of PLA2 allele and aspirin sensitivity (OR 0.7, 95% CI 0.52, 0.94; P = 0.02), but showed significant heterogeneity between studies (P = 0.02). This significance was lost when using the random effects model (OR 0.79, 95% CI 0.45, 1.38; P = 0.4).

### PlA1/A2 gene polymorphism and adverse cardiovascular outcomes

Eight studies^6–12,34^ including 4091 CAD patients were selected to investigate the association between PlA1/A2 gene polymorphism and adverse cardiovascular events. Details of the included studies are summarized in Table 2. Among the recruited studies, data on death was available in eight studies^6–12,34^; data on non-fatal MI was available in seven studies^6,8–12,34^; and data on TVR was available in five studies^6,8,10–12^.

**Table 2:** Characteristics of eligible studies referring to the association between PlA1/A2 gene polymorphism and adverse cardiovascular events.

Source	Number of patients (PlA1/PlA2)	Study design	Aspirin dosage (mg)	Mean follow-up	Outcomes (PlA1/PlA2)	
Addad F *et al*., 2010	188(104/84)	Cohort study	250	1 year	Death	(10/1)
Kastrati A *et al*., 2000	1759(1234/525)	Cohort study	200	1 month	Death	(4/3)
					MI	(51/24)
					TVR	(28/12)
Laule M *et al*., 1999	653(470/183)	Case-control study	100	1 month	MI	(8/4)
					Death	(1/0)
					TVR	(18/10)
Lopes NH *et al*., 2004	562(450/112)	Cohort study	UK	3 years	Death	(41/2)
					MI	(37/9)
					TVR	(44/2)
Syros G *et al*., 2009	200(144/56)	Cohort study	UK	1 year	Death	(2/2)
					MI	(1/0)
					TVR	(8/1)
Walter DH *et al*., 1997	318(255/63)	Cohort study	100–500	1 month	Death	(0/0)
					MI	(3/4)
Walter DH *et al*., 2001	324(253/71)	Cohort study	100	6 months	Death	(9/0)
					MI	(9/2)
					TVR	(83/30)
Wheeler GL *et al*., 2002	87(66/21)	Cohort study	UK	24 h	Death	(0/0)
					MI	(4/0)

MI, myocardial infarction; TVR, target vessel revascularization; UK, unknown.

**Figure 3:**
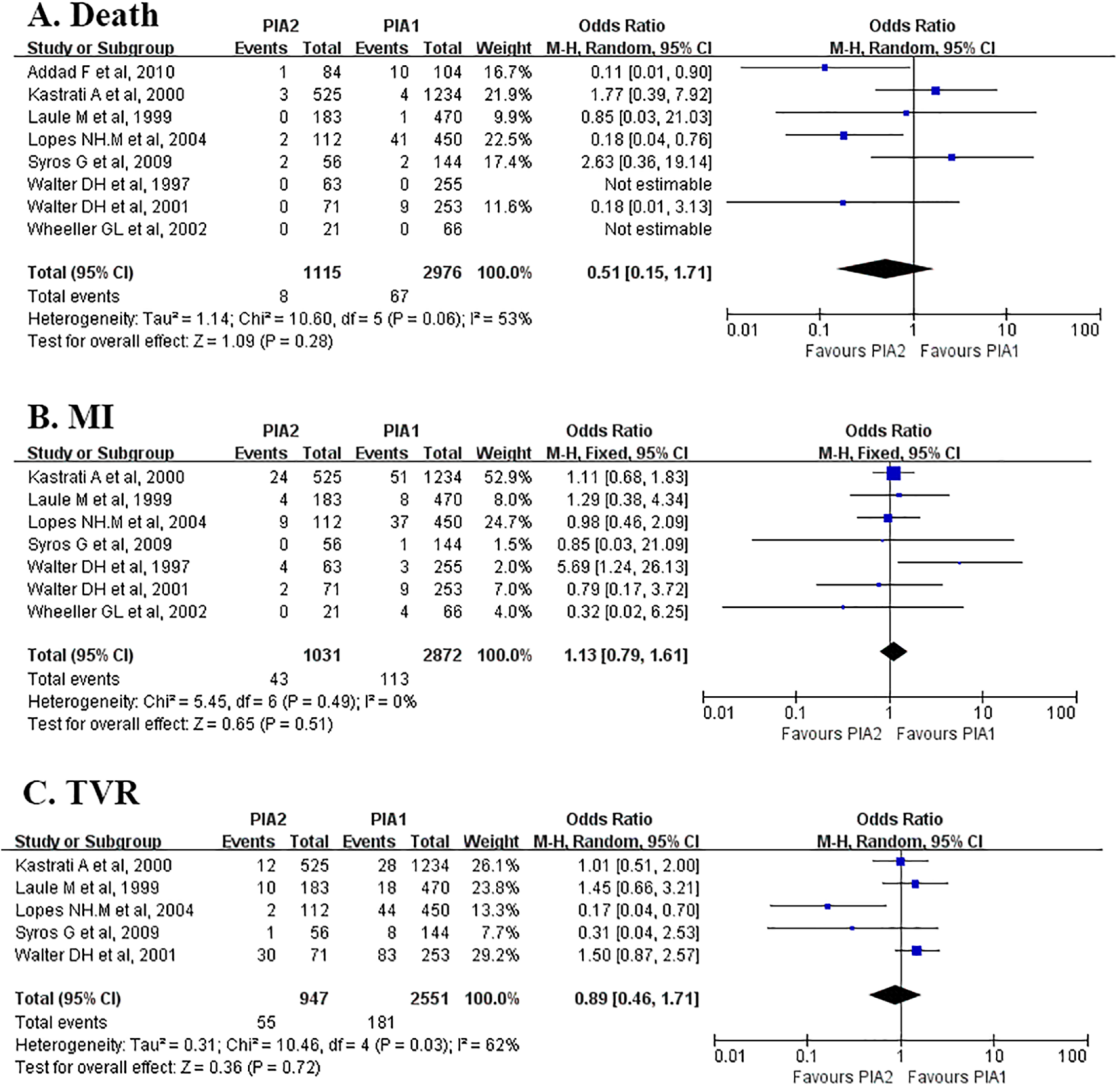
Association between PlA1/A2 polymorphism and clinical outcomes. Symbols and conventions are the same as in Fig. 2. This meta-analysis shows no significant change in the incidences of death, MI or TVR of patients carrying the PlA2 allele over those carrying PlA1 allele. The overall ORs of death, MI and TVR are 0.51(P = 0.28), 1.13 (P = 0.51) and 0.89 (P = 0.72) respectively. Note that the p-values mentioned here are the p-values for Z test. CI, confidence interval; MI, myocardial infarction; TVR, target vessel revascularization.

Meta-analysis showed that PlA2 gene carriers had similar risk of death compared with PlA1/A1^30^. The incidences of MI and TVR in PlA2 gene carriers (PlA1/A2 or PlA2/A2) were also similar to those with the wild genotype (PlA1/A1) with ORs of 1.13 (95% CI 0.79 to 1.61, P = 0.51) and 0.89(95% CI 0.46 to 1.61, P = 0.71) respectively (Fig. 3).

### Laboratory aspirin resistance and adverse cardiovascular outcomes

Eleven studies^13–17,35–40^ with 11857 aspirin-treated CAD patients were included to analyze the association between laboratory aspirin resistance and adverse cardiovascular outcomes. Details of included studies are summarized in Table 3, where mortality was reported in 10 studies^13–17,36–40^, ischemic stroke was reported in 5 studies^16,17,36–38^, and revascularization was reported in 4 studies^35,37–39^.

**Table 3:** Characteristics of eligible studies referring to the association between laboratory aspirin resistance and adverse cardiovascular outcomes.

Source	Number of patients (AR/AS)	Study design	Aspirin dosage (mg)	Method of assessment of lab AR	Definition of assessment of lab AR	Mean follow-up	Outcomes (AR/AS)	
Salah A *et al*., 2015	50(24/26)	Cohort study	150	LTA	PL_AA_ ≥ 20%	6 months	Death	(0/0)
							MI	(2/0)
Stone GW *et al*., 2013	8527(478/8049)	Cohort study	300–324	VerifyNow	>550ARU	1 years	Death	(3/253)
							MI	(7/143)
Li L *et al*., 1912	109(20/89)	Cohort study	100	CHRONO-LOG	>0Ω	1years	UK	UK
Glulmez O *et al*., 2008	114(27/87)	Cohort study	300	PFA-100	CEPI-CT < 165 s	1 week	UK	UK
Christiaens L *et al*., 2008	97(29/ 68)	Cohort study	160	PFA-100	CEPI-CT < 187 s	2.5 years	Death	(3/5)
							MI	(1/2)
Anderson K *et al*., 2002	71(25/46)	Cohort study	75–160	PFA-100	CEPI-CT < 196 s	4 years	MI	(3/4)
							TVR	(9/9)
Gum PA *et al*., 2003	326(17/309)	Cohort study	325	LTA	PL_ADP_ ≥ 70% and PL_AA_ ≥ 20%	679 days	Death	(2/15)
							MI	(1/12)
Kim HJ *et al*., 2002	220(39/181)	Cohort study	100	VerifyNow	≥550ARU	72 hours	Death	(0/1)
							MI	(1/12)
							TVR	(1/3)
Gori Am *et al*., 2016	1789(364/1425)	Cohort study	100–325	LTA	PL_AA_ ≥ 20%	2 years	Death	(35/54)
							MI	(9/32)
							TVR	(3/13)
Marcucci R *et al*., 2006	146(41/105)	Cohort study	100	PFA-100	CEPI-CT < 203 s	1 year	Death	(6/9)
							MI	(4/13)
							TVR	(8/4)
Foussas SG *et al*., 2009	496(121/375)	Cohort study	100/160/280/325	PFA-100	CEPI-CT < 193 s	1 year	Death	(28/36)

AR, aspirin resistance; AS, aspirin sensitive; LTA, light transmittance aggregometry; PL_AA_, AA-induced platelet aggregation; MI, myocardial infarction; ARU, aspirin reaction units;  UK, unknown; PFA-100, platelet function analyzer-100; CEPI-CT, collagen epinephrine-close time; TVR, target vessel revascularization; PL_ADP_, ADP-induced platelet aggregation.

**Figure 4:**
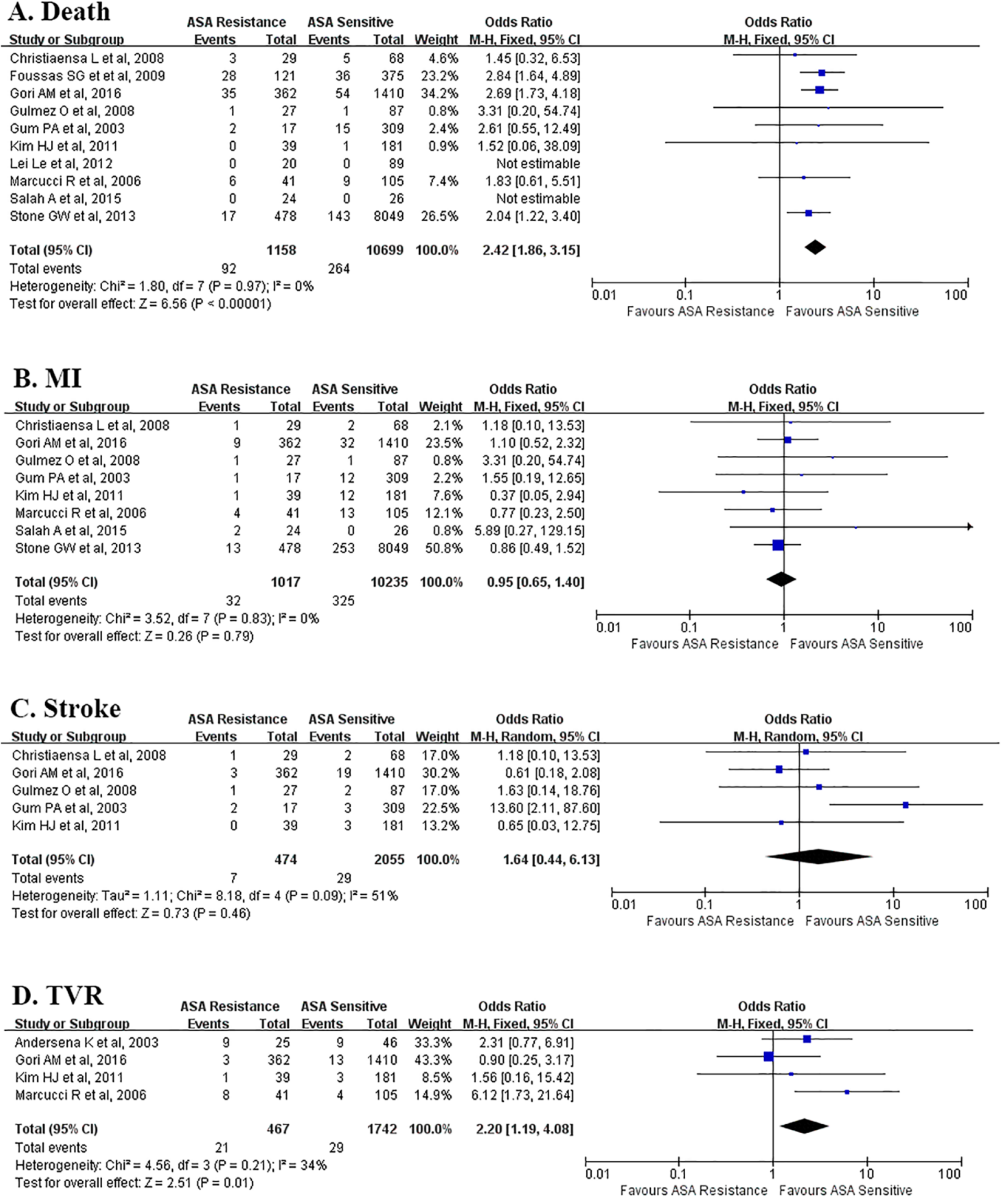
Association between laboratory aspirin resistance and clinical outcomes. Symbols and conventions are the same as in Fig. 2. This meta-analysis shows significant overall increase in the incidences of death and TVR of patients with AR over those without AR, while no significant change in the incidences of MI or stroke. The overall ORs in death, MI, stroke and TVR are 2.42 (P < 0.00001), 0.95 (P = 0.79), 1.64 (P = 0.46) and 2.2 (P = 0.01) respectively. Note that the p-values mentioned here are the p-values for Z test. CI, confidence interval; ASA, aspirin; MI, myocardial infarction; TVR, target vessel revascularization.

Meta-analysis showed that the laboratory aspirin resistance significantly increased the risk of all-cause death (OR = 2.42, 95% CI 1.86 to 3.15, I^2^ = 0%, P < 0.00001) and TVR (OR = 2.20, 95% CI 1.19 to 4.08, I^2^ = 13%, P = 0.01) (Fig. 4).

## Discussion

In this systematic review and meta-analysis we have found that: (1) there is no significant association between the PlA1/A2 polymorphism and aspirin resistance, or the PlA1/A2 polymorphism and worse clinical outcomes. (2) Laboratory aspirin resistance predicts all-cause death and TVR.

The PlA1/A2 gene polymorphism generated great interest since Weiss *et al*.^41^ first reported that PlA2 gene carriers presented a two-fold increase in risk of acute coronary syndromes. It has been clarified that PlA1/A2 encodes the platelet membrane glycoprotein IIIa which is integrated with glycoprotein IIb to form a complex. People with wild-type PlA1 have leucine at position 33 of mature glycoprotein IIIa, while those with point mutation PlA2 have proline at this position as a consequence of the substitution of cytosine for thymidine at position 1565 in exon 2 of the glycoprotein IIIa gene^42^. However, whether this mutation affects the anti-platelet effect of aspirin remains controversial.

By this meta-analysis, we found that PlA1/A2 polymorphism does not predict laboratory aspirin resistance. In fact, aspirin reduces the activation of platelets by irreversibly acetylating serine at position 529 of cyclooxygenase-1 (COX-1), and thereby reduces thromboxane A_2_ (TXA_2_) formation from the platelets^43^. So when aspirin effectively blocks the COX-1 channel, platelet aggregation would be effectively inhibited as a consequence of less TXA_2_ formation even in PlA2 carriers who have an increased activity of the GpIIb/IIIa receptor^44^. We suggest that PlA1/A2 polymorphism does not affect the anti-platelet effect of aspirin as aspirin has an upstream inhibitory effect on platelet aggregation, which may also account for its inability to predict the clinical outcomes.

Goodman *et al*.^45^ reported their meta-analysis which showed a genetic association between the PlA1/A2 molecular variant and aspirin resistance in healthy subjects who took aspirin alone. However, they failed to find significant association between carriage of the PlA2 allele and aspirin resistance in subjects with cardiovascular disease, which was consistent with our findings. In fact, in our study 51.1% of the CAD patients are on dual anti-platelet treatment with aspirin and a P2Y_12_ receptor antagonist, and it has been demonstrated that P2Y_12_ antagonists potentiate the inhibitory actions of PGI_2_, which would be converted to TXA_2_ ^46^. So it is possible that P2Y_12_ antagonists cause certain degree of platelet inhibition through blockage of TXA_2_ formation, and thereby obscuring the presence of aspirin resistance, as well as bias our study results. However, we believe that the efficacy of PlA1/A2 on aspirin resistance as well as the clinical outcomes would be too weak to be significant, should it exist.

Our study found that laboratory aspirin resistance predicted all-cause death and TVR, though the following studies reported negative results. Kim *et al*.^37^ included 220 patients who were planned to receive off-pump coronary artery bypass surgery (OPCAB), but it only recorded in-hospital clinical outcomes during the same admission period with relatively small sample size and very short follow-up intervals. This could lead to a study bias. The study conducted by Gulmez *et al*.^16^ was similar in this aspect.

Floyd *et al*. investigated the association between PlA1/A2 polymorphism of glycoprotein IIIa and the efficacy of aspirin by meta-analysis in 2014^47^, in which they included 14 papers^18,19,21,22,24–26,29–33,48,49^ published before 1 April 2013, and 1463 subjects who were homozygous for the PlA1 allele and 622 who carried the PlA2 allele were enrolled in their meta-analysis. In our study, however, we only included papers studying on patients with coronary artery disease, so we excluded 2 papers^48,49^ included in Floyd’s study, but recruited another 4 papers^20,23,27,28^ in the analysis. As a result, sixteen studies^18–33^ including 3077 CAD patients were enrolled to assess the association between PlA1/A2 gene polymorphism and aspirin resistance. To the best of our knowledge, this meta-analysis is the first to simultaneously investigate the relationship among PlA1/A2, aspirin resistance and clinical outcomes, which included the latest studies and recruited the largest study population, and would come out with the most updated, accurate and realistic results on this topic.

Although we adopted a random effect model in the process of this meta-analysis, the following limitations could not be avoided: (1) The recruited studies differed in genders and the duration of follow-up time, which might cause heterogeneities. If more data were available in the future, it would be preferable to perform a refined stratification analysis with data being adjusted for these factors. (2) The recruited studies differed in geographical areas, of which 4 were performed in Asia, 8 in America, and 12 in Europe. It was reported that the prevalence of the PlA2 allele is dependent on ethnicity, with a frequency of approximately 15 per 100 in Caucasian populations falling to 1 per 100 in Oriental populations^50,51^. If we have a detailed data on the ethnicity of each included patient, a stratification analysis on different ethnicity would be valuable to elucidate whether the results would be differ by ethnicity. (3) As pointed out by Floyd *et al*., mortality bias may attenuate or entirely obscure any true association^52^. Almost a third of individuals with a first major coronary event die out-of-hospital, and are not accounted for in the predominantly retrospective data presented in this meta- analysis^52^.

In conclusion, in aspirin-treated CAD patients, the laboratory aspirin resistance predicts all-cause death and TVR. However, the PlA1/A2 gene polymorphism predicts neither the laboratory aspirin response nor the clinical outcomes. Given this result, individualized anti-platelet treatment with the guidance of PlA1/A2 genetic testing may not be meaningful. This is in accordance with the 2018 ESC/EACTS guidelines on myocardial revascularization which recommended that genetic testing can not be recommended on a routine basis for tailoring and escalating dual anti-platelet treatment after stent implantation in all percutaneous coronary intervention (PCI)-treated patients^53^. However, as the results of laboratory platelet function test on individual aspirin response predict clinical outcomes, patients with aspirin resistance would benefit from intensified anti-platelet treatment.

## Supplementary information

Supplementary Figures
