# Supplementary material for: Association among PlA1/A2 gene polymorphism, laboratory aspirin resistance and clinical outcomes in patients with coronary artery disease: An updated meta-analysis
Source: Sci Rep. 2019 Sep 11;9:13177. doi: 10.1038/s41598-019-49123-y (PMC6739359; doi:10.1038/s41598-019-49123-y)
Supplement: Supplementary file 1 — Supplementary Figures [file 41598_2019_49123_MOESM1_ESM.pdf]

Association among PlA1/A2 gene polymorphism, laboratory aspirin resistance and clinical outcomes in patients with coronary artery disease: An updated meta-analysis

Jing Wang, Jie Liu, Yaqing Zhou, Fei Wang, Ke Xu, Deyu Kong, Jianling Bai, Jun Chen, Xiaoxuan Gong, Haoyu Meng, Chunjian Li

AR (LTA assay)

Study or Subgroup	PlA2 Events	PlA2 Total	PlA1 Events	PlA1 Total	Weight	Odds Ratio M-H, Fixed, 95% CI
Chunxiao L et al, 2011	0	0	8	152		Not estimable
Beiyun W et al, 2014	12	12	224	438	0.9%	23.89 [1.41, 405.94]
Lordkipanidze et al ,2011	6	139	2	52	5.0%	1.13 [0.22, 5.77]
Lev E et al,2007	4	31	8	89	6.4%	1.50 [0.42, 5.38]
Kranzhofer R et al, 2006	3	18	11	37	10.7%	0.47 [0.11, 1.97]
Jefferson et al ,2005	80	273	15	51	32.0%	0.99 [0.52, 1.92]
Papp E et al, 2005	41	87	78	198	45.0%	1.37 [0.82, 2.28]
**Total (95% CI)**		**560**		**1017**	**100.0%**	**1.35 [0.96, 1.91]**
Total events	146		346			

Heterogeneity: $Chi^2 = 6.94$, df = 5 (P = 0.22); $I^2 = 28\%$

Test for overall effect: Z = 1.70 (P = 0.09)

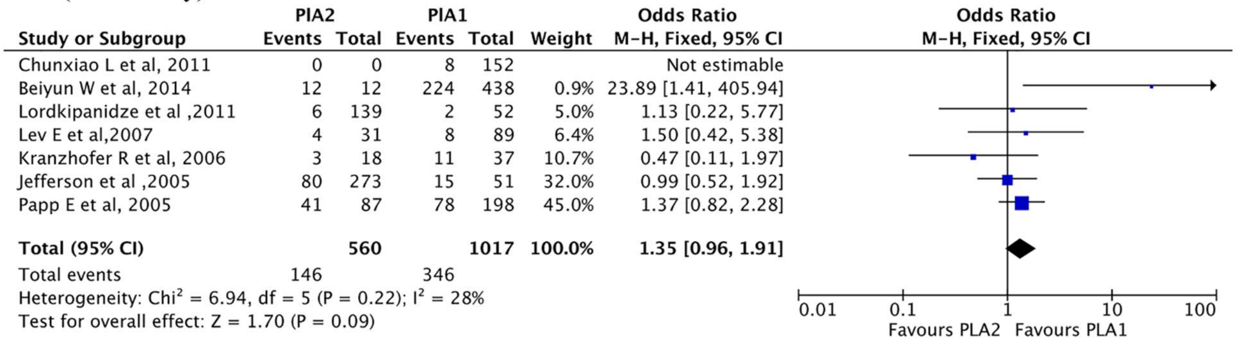

Supplementary Figure 1. Subgroup analysis of LTA.

Symbols and conventions are the same as in Figure 2. This meta-analysis shows no significant change in the incidence of LTA-determined AR of patients carrying the PlA2 allele over those carrying PlA1 allele. The overall OR is 1.35 (P=0.09). Note that the p-value mentioned here is the p-value for Z test. LTA, light transmittance aggregation.

AR (PFA-100 assay)

Study or Subgroup	PLA2 Events	PLA2 Total	PLA1 Events	PLA1 Total	Weight	Odds Ratio M-H, Fixed, 95% CI
Pamukcu et al ,2010	5	6	25	41	1.0%	3.20 [0.34, 29.96]
Godeneche et al ,2009	6	26	4	56	1.8%	3.90 [0.99, 15.29]
Bernardo E et al ,2006	10	26	15	50	5.8%	1.46 [0.54, 3.94]
Pamukcu B et al ,2005	7	21	36	73	9.9%	0.51 [0.19, 1.42]
Macchi L et al ,2003	4	32	25	66	13.2%	0.23 [0.07, 0.75]
Abderrazek F et al ,2010	28	84	53	104	29.2%	0.48 [0.27, 0.87]
Kunicki et al ,2009	32	151	79	296	39.0%	0.74 [0.46, 1.18]
**Total (95% CI)**		**346**		**686**	**100.0%**	**0.70 [0.52, 0.94]**
Total events	92		237			

Heterogeneity: $Chi^2 = 15.29$, df = 6 (P = 0.02); $I^2 = 61\%$

Test for overall effect: Z = 2.39 (P = 0.02)

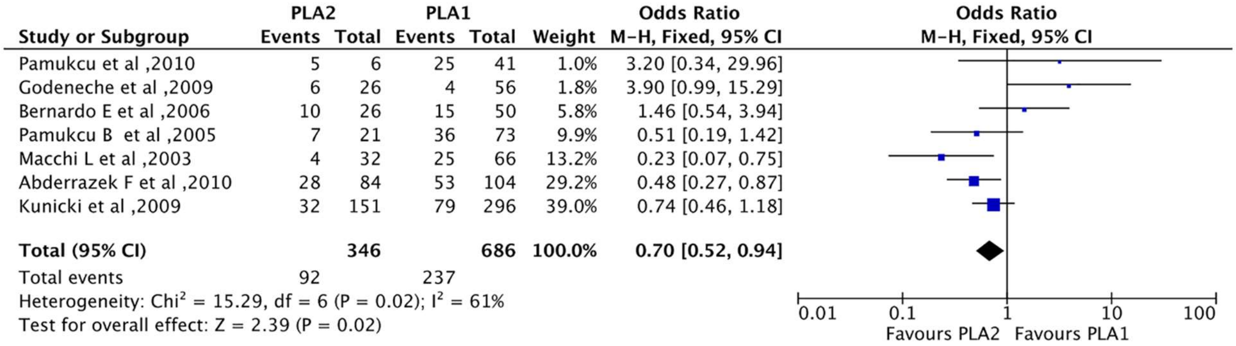

Supplementary Figure 2. Subgroup analysis of PFA-100.

Symbols and conventions are the same as in Figure 2. This meta-analysis shows a significant overall decrease in the incidence of PFA-determined AR of patients carrying the PlA2 allele over those carrying PlA1 allele. The overall OR is 0.7 (P=0.02). Note that the p-value mentioned here is the p-value for Z test. PFA-100, platelet function analyzer 100.
